# Predictive value of thrombus volume for recanalization in stent retriever thrombectomy

**DOI:** 10.1038/s41598-017-16274-9

**Published:** 2017-11-21

**Authors:** Jang-Hyun Baek, Joonsang Yoo, Dongbeom Song, Young Dae Kim, Hyo Suk Nam, Byung Moon Kim, Dong Joon Kim, Hye Sun Lee, Ji Hoe Heo

**Affiliations:** 1 Department of Neurology, Yonsei University College of Medicine, Seoul, Republic of Korea; 2 Department of Radiology, Yonsei University College of Medicine, Seoul, Republic of Korea; 3 Biostatistics Collaboration Unit, Yonsei University College of Medicine, Seoul, Republic of Korea; 4 Department of Neurology, National Medical Centre, Seoul, Republic of Korea; 5 Department of Neurology, Keimyung University College of Medicine, Daegu, Republic of Korea

## Abstract

This retrospective study investigated whether the volume or density of the thrombus is predictive of recanalization in stent retriever (SR) treatment. Consecutive patients treated with SR thrombectomy as the first endovascular modality were enrolled. The thrombus volume and density were measured on thin-section noncontrast computed tomography using 3-dimensional software. The patients were grouped by recanalization status and the number of SR passes. Among 165 patients, recanalization was achieved with the first pass in 68 (50.0%), 2–3 passes in 43 (31.6%), and ≥4 passes in 25 (18.4%) patients. The thrombus volume was smaller in patients with (107.5 mm^3^) than without (173.7 mm^3^, p = 0.025) recanalization, and tended to be larger with increasing number of passes (p for trend = 0.001). The thrombus volume was an independent predictor of first-pass recanalization (odds ratio 0.93 per 10 mm^3^, 95% confidence interval 0.89–0.97). However, the thrombus density was not associated with recanalization success. Recanalization within 3 passes was associated with a favorable outcome. In conclusion, the thrombus volume was significantly related to recanalization in SR thrombectomy. Measuring the thrombus volume was particularly predictive of first-pass recanalization, which was associated with a higher likelihood of a favorable outcome.

## Introduction

Thrombectomy with a stent retriever (SR) is recommended as a first-line endovascular treatment, with the highest level of evidence^1,2^. Recent meta-analyses have shown that recanalization could be achieved in up to 80% of patients using an SR^3,4^. However, only 54% of patients experienced favorable outcomes, which suggests that recanalization is futile in many patients.

The chance of futile recanalization increases with the number of passes. However, the optimal number of passes with the SR to avoid futile recanalization is uncertain. Moreover, responses to recanalization treatment may differ according to the characteristics of the thrombus, such as volume and physical properties. A small thrombus may be easily retrieved with an SR, requiring only a few passes and yielding a greater chance of a favorable outcome. However, few studies have investigated the association between thrombus characteristics and recanalization in view of the number of passes^5,6^.

We have previously shown that the volume and density of the thrombus can be measured quantitatively on thin-section noncontrast computed tomography (NCCT) scans by means of software^7,8^. This study aimed to investigate whether the volume or density of the thrombus measured on thin-section NCCT is predictive of recanalization after intra-arterial recanalization therapy (IART) with an SR. We also investigated whether there is an association between the volume or density of the thrombus and the number of SR passes to achieve recanalization.

## Results

**Figure 1:**
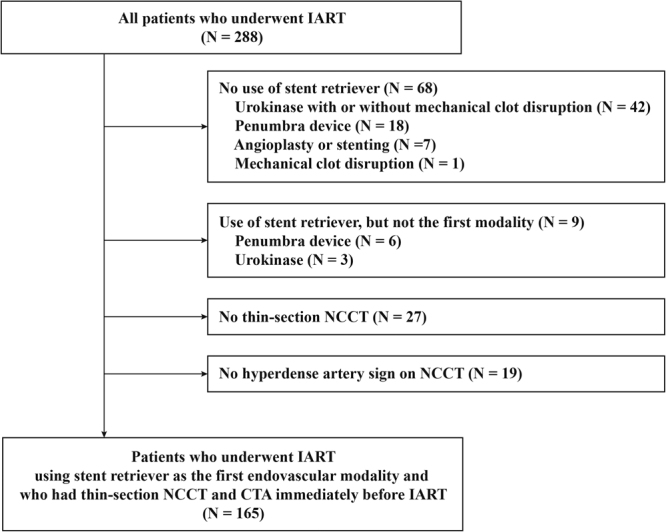
Patient selection flow chart. IART, intra-arterial recanalization therapy; NCCT, non-contrast computed tomography; CTA, computed tomography angiography.

Of 288 patients who received IART during the study period, 211 patients were treated with SR thrombectomy as the first endovascular modality (Fig. 1). After excluding patients without thin-section NCCT (n = 27) or without hyperdense arteries on NCCT (n = 19), 165 patients were finally included.

For the 165 patients, the mean age was 70.3 ± 11.2 years and 82 (49.7%) were men. The median thrombus volume was 119.6 mm^3^ (interquartile range [IQR] 53.9–205.4), and the median corrected Hounsfield unit (HU) of the thrombus was 53.3 (IQR 48.8–58.2). The most common occlusion site was the middle cerebral artery M1 segment, followed by the internal carotid artery (Table 1).

**Table 1:** Occlusion site and thrombus characteristics.

Occlusion site	Number of patients	Volume, mm^3^	Density, corrected HU
Anterior circulation			
Internal carotid artery	46 (27.9)	211.2 [136.8–287.9]	56.5 [52.0–60.6]
Middle cerebral artery, M1	81 (49.1)	104.8 [59.2–151.1]	52.6 [48.1–57.0]
Middle cerebral artery, M2	14 (8.5)	33.2 [27.6–51.0]	52.2 [49.1–56.8]
Posterior circulation			
Basilar artery	23 (13.9)	62.9 [39.2–156.3]	52.2 [49.4–56.7]
Posterior cerebral artery	1 (0.6)	9.31	65.9
Total	165 (100)	119.6 [53.9–205.4]	53.3 [48.8–58.2]

Values are number (%) or median [interquartile range]. HU, Hounsfield unit.

### Association between thrombus volume and successful recanalization

**Figure 2:**
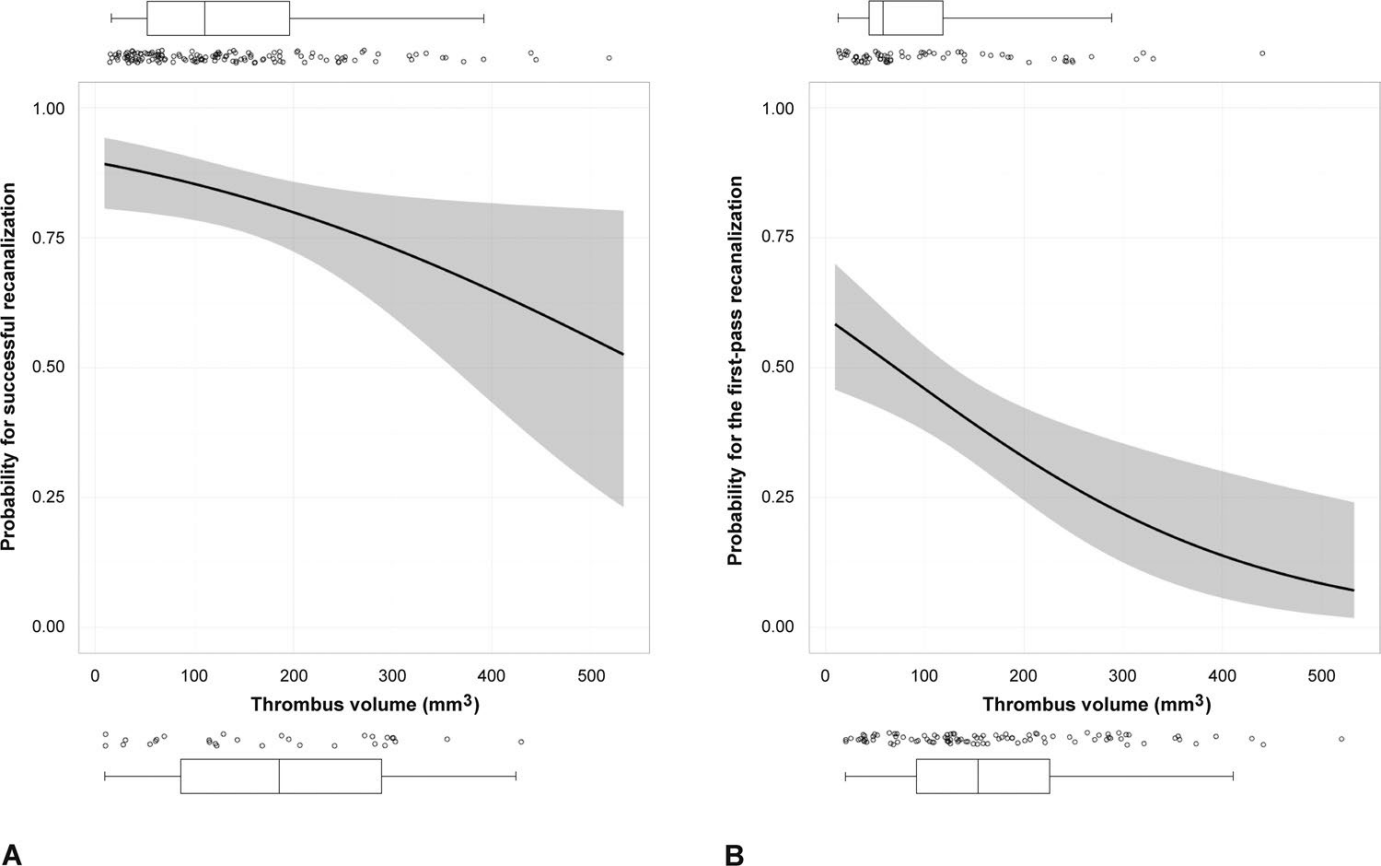
Logistic regression curves representing the probability of recanalization depending on thrombus volume. Increasing thrombus volume was associated with a significantly decreased probability of (**A**) successful recanalization and (**B**) the first-pass recanalization. Dots represent the thrombus volume of each case. The boxplot represents the median and interquartile range.

Successful recanalization was achieved in 136 of 165 patients (82.4%). The median thrombus volume was smaller in patients with (107.5 mm^3^) than in those without successful recanalization (173.7 mm^3^, p = 0.025) (Table 2). On multivariable analysis, the use of a balloon-guiding catheter (BGC) and the thrombus volume were independent factors for successful recanalization (Table 2 and Fig. 2A). The thrombus density did not differ statistically significantly between patients with and those without successful recanalization.

**Table 2:** Factors associated with a successful recanalization.

	Univariable analysis			Multivariable analysis		
	With successful recanalization (n = 136)	Without successful recanalization (n = 29)	P	OR	(95% CI)	P
Age, years	69.9 ± 10.7	72.0 ± 13.3	0.357	0.99	(0.95–1.03)	0.577
Sex, men	69 (50.7)	13 (44.8)	0.563	1.27	(0.54–2.97)	0.585
Hypertension	95 (69.9)	23 (79.3)	0.306			
Diabetes	43 (31.6)	9 (31.0)	0.951			
Smoking	21 (15.4)	2 (6.9)	0.375			
Hypercholesterolemia	25 (18.4)	5 (17.2)	0.885			
Initial NIHSS score	16.0 [13.0–9.0]	18.0 [15.0–21.0]	0.082	0.97	(0.90–1.05)	0.486
Atrial fibrillation	85 (62.5)	19 (65.5)	0.760			
Occlusion sites			0.274			
Internal carotid artery	35 (25.7)	11 (37.9)				
Middle cerebral artery	82 (60.3)	13 (44.8)				
Posterior circulation	19 (14.0)	5 (17.3)				
Use of intravenous tPA	55 (40.4)	9 (31.0)	0.345			
Use of BGC	75 (55.1)	9 (31.0)	0.018	2.59	(1.08–6.26)	0.034
Size of stent retriever			0.964			
4/15	4 (2.9)	1 (3.4)				
4/20	126 (92.7)	27 (93.2)				
6/30	6 (4.4)	1 (3.4)				
Time from onset to puncture, min	202.5 [150.0–323.2]	229.0 [160.0–411.0]	0.292			
Thrombus density, corrected HU	52.8 [48.6–57.7]	54.7 [52.1–60.6]	0.175			
Thrombus volume, mm^3^	107.5 [50.8–183.8]	173.7 [73.2–289.2]	0.025	0.96^a^	(0.93–0.98)	0.040
Favorable outcome	72 (52.9)	4 (13.8)	<0.001			

Values are mean ± standard deviation, number (%), or median [interquartile range]. ^a^Odds ratio per 10 mm^3^ of thrombus volume. OR, odds ratio; CI, confidence interval; NIHSS, National Institutes of Health Stroke Scale; tPA, tissue plasminogen activator; BGC, balloon-guiding catheter; HU, Hounsfield unit.

### Association between thrombus volume and the number of stent retriever passes

Of 136 patients with successful recanalization, recanalization was achieved with the first pass of the SR in 68 patients (50.0%), in 2 or 3 passes in 43 patients (31.6%), and in ≥4 passes in 25 patients (18.4%) (Table 3). The thrombus volume tended to be larger as the number of passes for recanalization increased (p for trend = 0.001). The median thrombus volume in the first-pass recanalization group was 63.9 mm^3^, which was markedly smaller than in the other groups. As the number of passes increased, the time from puncture to recanalization also increased (p = 0.001).

**Table 3:** Comparison of variables according to the number of stent retriever passes.

	First-pass recanalization (n = 68)	2- or 3-Pass recanalization (n = 43)	≥4-Pass recanalization (n = 25)	No recanalization (n = 29)	P^a^	P for trend^b^
Age, years	69.4 ± 11.5	71.7 ± 9.4	68.2 ± 10.5	72.0 ± 13.3	0.449	0.588
Sex, men	31 (45.6)	23 (53.5)	15 (60.0)	13 (44.8)	0.564	0.724
Hypertension	45 (66.2)	32 (74.4)	18 (72.0)	23 (79.3)	0.573	0.203
Diabetes	22 (32.4)	11 (25.6)	10 (40.0)	9 (31.0)	0.668	0.849
Smoking	10 (14.7)	4 (9.3)	7 (28.0)	2 (6.9)	0.135	0.828
Hypercholesterolemia	16 (23.5)	6 (14.0)	3 (12.0)	5 (17.2)	0.478	0.303
Initial NIHSS score	15.0 [11.8–19.0]	17.0 [13.0–20.0]	17.0 [14.0–22.0]	18.0 [15.0–21.0]	0.054^‡^	0.012
Atrial fibrillation	41 (60.3)	29 (67.4)	15 (60.0)	19 (65.5)	0.861	0.714
Occlusion sites					0.423	0.158
Internal carotid artery	15 (22.1)	10 (23.3)	10 (40.0)	11 (37.9)		
Middle cerebral artery	42 (61.8)	28 (65.1)	12 (48.0)	13 (44.8)		
Posterior circulation	11 (16.1)	5 (11.6)	3 (12.0)	5 (17.3)		
Use of intravenous tPA	29 (42.7)	19 (44.2)	7 (28.0)	9 (31.0)	0.405	0.162
Use of BGC	40 (58.8%)	25 (58.1%)	10 (40.0%)	9 (31.0)	0.039^‡^	0.006
Size of stent retriever					0.232	0.114
4/15	1 (1.5)	1 (2.3)	2 (8.0)	1 (3.4)		
4/20	66 (97.0)	40 (93.0)	20 (80.0)	27 (93.2)		
6/30	1 (1.5)	2 (4.7)	3 (12.0)	1 (3.4)		
Time from onset to puncture, min	190.0 [150.0–313.0]	210.0 [153.0–311.0]	205.0 [150.0–372.0]	229.0 [160.0–411.0]	0.571	0.201
Time from puncture to recanalization, min	45.0 [34.0–64.5]	65.0 [55.0–99.0]	120.0 [82.0–146.0]	NA	0.001^*^	0.001
Thrombus density, corrected HU	54.0 [50.1–59.4]	51.0 [46.8–55.9]	53.9 [47.7–60.1]	54.7 [52.2–60.6]	0.050^‡^	0.481
Thrombus volume, mm^3^	63.9 [37.9–146.6]	122.7 [74.6–210.1]	157.1 [108.9–187.5]	173.7 [73.2–289.2]	0.001^†^	0.001

Values are mean ± standard deviation, number (%), or median [interquartile range]. ^a^Analysis of variance, Kruskal-Wallis test, or chi-square or Fisher’s exact test. ^b^Analysis of variance for trend, Jonckheere-Terpstra test, or Mantel-Haenszel chi-square test. Multiple comparisons were calculated by Bonferroni-corrected Dunn procedure or Bonferroni-corrected chi-square test for initial NIHSS score, use of BGC, time from puncture to recanalization, thrombus density, and thrombus volume. *First-pass recanalization vs. 2- or 3-pass recanalization, P < 0.001; First-pass recanalization vs. ≥4-pass recanalization, P < 0.001; 2- or 3-pass recanalization vs. ≥4-pass recanalization, P < 0.001. ^†^First-pass recanalization vs. ≥4-pass recanalization, P = 0.016; First-pass recanalization vs. no recanalization, P = 0.006. ^‡^>0.05 on multiple comparisons. NIHSS, National Institutes of Health Stroke Scale; tPA, tissue plasminogen activator; BGC, balloon-guiding catheter; HU, Hounsfield unit; NA, not applicable.

We determined factors that were associated with recanalization according to the number of SR passes. On univariable multinomial logistic regression analysis (see Supplementary Table S1) and multivariable multinomial logistic regression analysis, the thrombus volume was found to be an independent predictor for first-pass recanalization (OR 0.94 per 10 mm^3^, 95% CI 0.89–0.98; p = 0.005) (Table 4 and Fig. 2B). The use of BGC was an independent predictor for successful recanalization within 3 passes.

**Table 4:** Multivariable multinomial logistic regression analysis for the number of stent retriever passes.

	First-pass recanalization		2- or 3-Pass recanalization		≥4-Pass recanalization	
	OR (95% CI)	P	OR (95% CI)	P	OR (95% CI)	P
Age, per 1 year	0.99 (0.94–1.04)	0.646	1.00 (0.95–1.05)	0.907	0.97 (0.92–1.02)	0.236
Sex, men	1.00 (0.39–2.57)	0.999	1.39 (0.52–3.73)	0.511	1.70 (0.56–5.18)	0.353
Initial NIHSS score	0.94 (0.86–1.02)	0.151	0.98 (0.89–1.07)	0.629	1.03 (0.94–1.13)	0.543
Use of BGC	3.19 (1.21–8.39)	0.019	2.99 (1.09–8.20)	0.034	1.49 (0.47–4.73)	0.499
Thrombus volume, per 10 mm^3^	0.94 (0.89–0.98)	0.005	0.97 (0.93–1.02)	0.241	0.99 (0.94–1.03)	0.619

Odds ratio was calculated over no recanalization group. OR, odds ratio; CI, confidence interval; NIHSS, National Institutes of Health Stroke Scale; BGC, balloon-guiding catheter.

As the thrombus volume was the only predictive factor for first-pass recanalization, we determined the cutoff thrombus volume that predicted first-pass recanalization, and found it to be 76.4 mm^3^ (sensitivity 55.9%, specificity 74.4%, and positive-predictive value 62.3%). The discrimination between the first-pass recanalization and other passes, according to this cutoff thrombus volume, was statistically significant (generalized c-index 0.630 with 95% CI 0.566–0.695; p < 0.001).

### Factors associated with functional outcomes

Of the 165 patients, 76 (46.1%) had a favorable outcome. Favorable outcomes were achieved in 64.7% (44 of 68) of the first-pass recanalization group and in 46.5% (20 of 43) of the 2- or 3-pass recanalization group. However, only 32.0% (8 of 25) of the ≥4-pass recanalization group and 13.8% (4 of 29) of the failure group showed favorable outcomes. A younger age, lower initial NIHSS score, and recanalization within 3 passes were independent predictors of a favorable outcome (Table 5). Although patients with unfavorable outcome had higher thrombus density and larger thrombus volume in univariable analysis, no associations were found between them in multivariable analysis, which suggests that they were not independent factors for functional outcome.

**Table 5:** Factors associated with a favorable outcome.

	Univariable analysis			Multivariable analysis		
	Unfavorable outcome (n = 89)	Favorable outcome (n = 76)	P	OR	(95% CI)	P
Age, years	72.9 ± 10.5	67.3 ± 11.2	0.001	0.95^a^	(0.92–0.99)	0.010
Sex, men	41 (46.1)	41 (53.9)	0.313	1.17	(0.54–2.52)	0.692
Hypertension	65 (73.0)	53 (69.7)	0.640			
Diabetes	30 (33.7)	22 (28.9)	0.512			
Smoking	9 (10.1)	14 (18.4)	0.125			
Hypercholesterolemia	11 (12.4)	19 (25.0)	0.036	2.55	(0.90–7.20)	0.077
Initial NIHSS score	18.0 [16.0–21.0]	14.0 [10.0–18.2]	0.001	0.88	(0.81–0.95)	0.001
Atrial fibrillation	61 (68.5)	43 (56.6)	0.123			
Occlusion sites			0.012			
Internal carotid artery	32 (36.0)	14 (18.4)		Reference		
Middle cerebral artery	42 (47.2)	53 (69.7)		1.83	(0.69–4.88)	0.226
Posterior circulation	15 (16.8)	9 (11.9)		1.41	(0.30–6.63)	0.665
Use of intravenous tPA	31 (34.8)	33 (43.4)	0.259			
Use of BGC	39 (43.8)	45 (59.2)	0.049	1.53	(0.65–3.61)	0.330
Time from onset to puncture, min	225.0 [154.0–320.0]	199.0 [149.2–340.0]	0.412			
Time from onset to recanalization, min^b^	323.5 [239.8–396.2]	278.0 [202.0–390.8]	0.155			
Thrombus density, corrected HU	55.6 [50.8–60.5]	52.1 [47.2–56.6]	0.004	0.97	(0.92–1.01)	0.169
Thrombus volume, mm^3^	138.5 [65.4–222.1]	84.8 [46.9–170.7]	0.014	0.99^c^	(0.96–1.04)	0.804
Endovascular outcome			0.001			
First-pass recanalization	24 (27.0)	44 (57.9)		9.25	(2.39–35.8)	0.001
2- or-3-Pass recanalization	23 (25.8)	20 (26.3)		4.94	(1.18–20.6)	0.028
≥4-Pass recanalization	17 (19.1)	8 (10.5)		3.10	(0.63–15.2)	0.162
No recanalization	25 (28.1)	4 (5.3)		Reference		

Values are mean ± standard deviation, number (%), or median [interquartile range]. ^a^Odds ratio per 1 year. ^b^Values for 136 patients with successful recanalization. ^c^Odds ratio per 10 mm^3^. OR, odds ratio; CI, confidence interval; NIHSS, National Institutes of Health Stroke Scale; tPA, tissue plasminogen activator; BGC, balloon-guiding catheter; HU, Hounsfield unit.

## Discussion

The thrombus is the ultimate target of recanalization treatment. The volume and composition of the thrombus may affect the response to recanalization treatment. This study showed that the thrombus volume was significantly related to successful recanalization with an SR. Previous studies have shown inconsistent findings in terms of the association between the thrombus burden and recanalization after mechanical thrombectomy by means of an SR^6,9–15^. The inconsistency between the studies might be partly ascribed to differences in the method used to measure thrombus burden and the number of patients^16^. Thrombus length and clot burden score could be surrogates for clot burden. It is very difficult to measure thrombus length on NCCT images without the aid of specific software. Therefore, the measurement is usually based on CT angiography (CTA). Determination of thrombus length on CTA is based on the presence of contrast opacification. This method indirectly measures the thrombus length as the portion lacking contrast opacification is assumed to be thrombi, and is influenced by the degree of backflow from the collateral circulation^17^. Furthermore, for curved or branched arteries and arteries with change in caliber, measurement of thrombus length is not easy on 2-dimensional images, and might not accurately represent the actual thrombus burden. The thrombus volume measured using 3-dimensional software in this study may provide direct, accurate, and reliable information on thrombus burden, irrespective of angioarchitecture and collaterals.

We also compared the thrombus volume and recanalization according to the number of passes for recanalization. Although there was a trend that the number of passes needed for recanalization increased as the volume of the thrombus increased, the association between the volume and recanalization was significant only in the first-pass recanalization group. This first-pass recanalization group had a much smaller thrombus and a markedly higher chance of a favorable outcome than did the other groups. The cutoff volume for predicting first-pass recanalization was calculated as 76.4 mm^3^ in this study population. However, this cutoff value could differ according to the study population and the use of different mechanical devices. In this study, BGC was also an independent predictor for successful recanalization and recanalization within 3 passes. BGC was known to be effective for reducing distal emboli during mechanical thrombectomy^18^, and for achieving more recanalization with fewer SR passes^19,20^.

We also investigated whether thrombus density is predictive of recanalization after SR treatment. This study showed no association between the average thrombus density and recanalization. Previous studies on patients undergoing endovascular treatment showed inconsistent findings in terms of the association between thrombus density and recanalization^6,10,21^. The thrombus density on CT scan may represent the constituents of the thrombus, in that the HU is high in red blood cell-rich regions and low in fibrin- or platelet-rich regions^22^. However, the distribution of each constituent within the thrombus is neither even, nor homogeneous, among thrombi^23^. Thus, defining the region of interest (ROI) might be important for accurate measurement of thrombus density, and, ideally, the entire thrombus should be included for averaging. In previous studies, the ROI was defined manually by outlining the margin of the thrombus or drawing small circles within the thrombus^6,10,24–26^. The former approach might underestimate the density, because the low-density areas in the periphery or outside of thrombus could be included during manual outlining of the thrombus margin^16^. On the contrary, the latter approach might overestimate the density by including only high-density areas within the thrombus^16^. In this study, pixels of almost the entire thrombus could be averaged as the ROI was defined automatically, based on HU, without the risk of a selection bias, using 3-dimensional software^8,27,28^. Besides the average density, the age and architecture of the thrombus might also affect the physical properties of the thrombus. These factors may also be associated with the retrieval of the thrombus using an SR; however, they cannot be assessed by simple measurement of the average HU of the thrombus.

This study has several limitations. First, this study involved retrospective analysis of data. The use of mechanical devices and the number of passes were not controlled. Thus, this study is not free from selection bias. Second, this study included patients who were treated with an SR. Therefore, the interpretation of our results should be limited to the use of an SR, because the association between the thrombus characteristics and recanalization might differ among the mechanical devices used. Third, patients without hyperdense artery signs were not included, because thrombus volume could not be measured in these cases. The absence of a hyperdense artery sign may be associated with the presence of thrombi with very low HU or non-embolic infarctions, such as intracranial artery stenosis^29,30^. Fourth, this study included patients who were treated with IV tPA before the endovascular treatment, which is currently the standard treatment. The guideline recommends that patients eligible for IV tPA should receive IV tPA even if endovascular treatments are being considered^31^. Although the frequency of recanalization was not different between patients with bridging IV thrombolysis and those with direct endovascular treatment in this study, IV tPA treatment might affect on the thrombus composition and structure. This should be considered for the interpretation of findings in this study. Finally, although the benefit of the use of an SR was observed when recanalization could be achieved within 3 passes in this study population, the effective number of passes may be different in other clinical settings, with different onset-to-recanalization time, because the benefit is time-dependent^32^.

## Conclusions

Our findings suggest that thin-section NCCT-based thrombus imaging can be useful for the prediction of successful recanalization, in that the volume of the thrombus was significantly related to recanalization after the use of an SR. Measuring the volume of the thrombus may be particularly useful for predicting first-pass recanalization after SR treatment, which can be achieved in many patients and has higher chances of achieving favorable clinical outcomes. Our findings also suggest that measuring the volume of the thrombus may assist the neurointerventionist to select the appropriate device for thrombectomy, for example, larger thrombi need longer or larger SRs, or aspiration devices.

## Methods

### Study population

We retrospectively analyzed the data of a prospective cohort that had been studied to investigate the predictive value of thrombus imaging based on thin-section NCCT^27,28^. The subjects of this study were consecutive patients with acute intracranial artery occlusion who underwent IART from September 2010 to August 2015. We included patients who were treated with SR thrombectomy as the first endovascular modality, and who had undergone thin-section (1-mm or 1.2-mm thickness) NCCT immediately before the IART. A Solitaire (Medtronic, Dublin, Ireland) stent retriever was used exclusively for endovascular treatment. Imaging protocols and IART procedures are described in detail in the Supplementary Methods.

The prospective cohort and the protocol of this study were approved by the Institutional Review Board of Severance Hospital, Yonsei University Health System. All methods were performed in accordance with the relevant guidelines and regulations. We obtained written informed consent from the patients or their qualified next of kin. The need to obtain informed consent for this specific study was waived due to its retrospective nature.

### Measurements of thrombus volume and Hounsfield units

The volume and density (HU) of the thrombus were measured on thin-section NCCT in a semi-automatic manner by using 3-dimensional software (Xelis; Infinitt, Seoul, Korea), as published previously^27,28^. For patients who received IV tPA, the volume and density of thrombus were assessed on follow-up NCCT images, which were taken just before endovascular treatment. Briefly, after the identification of the clot based on the pixel segmentation at a threshold between 50 and 100 HU, an ROI was drawn within any portion of the thrombus by using a brush tool or by a simple click. Then, by clicking the “dilate” button, automatic pixel dilation and region growing were performed to the margin of the thrombus at a threshold between 40 and 100 HU. It was unnecessary to define the margin of the thrombus manually. After this process, the volume and density of thrombus were automatically calculated and presented in table format. The calculated HUs were standardized to the HU value of the contralateral M1 segment of the middle cerebral artery (corrected HU; see Supplementary Methods)^27^. Although beam-hardening artifacts may occur in the skull base, which may disturb the assessment of thrombi in the internal carotid artery or basilar artery, the artifact was minimized using a reconstruction algorithm and post-imaging processing. In fact, no case in this study population had such an artifact.

Two investigators who were blinded to the clinical details measured the volume and density of the thrombus independently. The inter-rater agreements were excellent for both volume (interclass coefficient 0.989) and density (0.950).

### Outcomes

Functional outcome was determined using the modified Rankin Scale (mRS) score at 3 months after stroke onset. A favorable outcome was defined as an mRS 0–2. Successful recanalization was defined as the modified Thrombolysis In Cerebral Infarction (mTICI) 2b or 3 on the final angiogram during IART^33^. mTICI 2b was defined as antegrade partial perfusion of half or greater of the downstream ischemic territory, and mTICI 3 was defined as antegrade complete perfusion of the downstream ischemic territory. All reperfusion grades were determined with consensus from stroke neurologists, neurointerventionists, and neuroradiologists during the regular stroke conference. The basic principle of mTICI grading was also applied to posterior circulation stroke. Based on the specific target arterial lesion and its relevant target downstream territory, the ratio of final reperfusion area was estimated^34^. We divided the patients into 4 groups based on the recanalization status and the number of SR passes for achieving recanalization, which included the first-pass recanalization, 2- or 3-pass recanalization, ≥4-pass recanalization, and no recanalization.

### Statistical analyses

We compared demographic and clinical variables, and the volume and HU of the thrombus between the groups, using analysis of variance, the Kruskal-Wallis test, chi-square test, or Fisher’s exact test, as appropriate. We also performed analysis of variance for trend, the Jonckheere-Terpstra test, or the Mantel−Haenszel test for trend analysis. To determine independent factors for predicting recanalization, multivariable binary or multinomial logistic regression was performed by entering age, sex, and variables of p < 0.10 on univariable analyses. The diagnostic performances for predicting recanalization were calculated using the cutoff values for each histogram parameter according to the Youden index. The generalized c-index and 95% CI were calculated using the bootstrapping method with resampling of 1,000 times. The factors associated with the functional outcome were compared using Student’s *t*-test, Wilcoxon’s signed-rank test, chi-square test, or Fisher’s exact test. We also performed multivariable binary logistic regression analysis to determine independent factors for a favorable outcome.

All statistical analyses were performed with R software package version 3.2.2 (http://www.R-project.org) or SAS (version 9.2; SAS Inc., Cary, NC, USA). P < 0.05 was considered statistically significant.

## Electronic supplementary material

Supplementary Data
