# Supplementary material for: Predictive value of thrombus volume for recanalization in stent retriever thrombectomy
Source: Sci Rep. 2017 Nov 21;7:15938. doi: 10.1038/s41598-017-16274-9 (PMC5698357; doi:10.1038/s41598-017-16274-9)
Supplement: Supplementary file 1 — Supplementary Data [file 41598_2017_16274_MOESM1_ESM.pdf]

Predictive value of thrombus volume for recanalization in stent retriever thrombectomy

Jang-Hyun Baek[1,4], Joonsang Yoo[1,5], Dongbeom Song[1], Young Dae Kim[1]
Hyo Suk Nam[1], Byung Moon Kim[2], Dong Joon Kim[2], Hye Sun Lee[3], and Ji Hoe Heo[1,*]

[1]Department of Neurology, Yonsei University College of Medicine, Seoul, Republic of Korea
[2]Department of Radiology, Yonsei University College of Medicine, Seoul, Republic of Korea
[3]Biostatistics Collaboration Unit, Yonsei University College of Medicine, Seoul, Republic of Korea
[4]Department of Neurology, National Medical Centre, Seoul, Republic of Korea
[5]Department of Neurology, Keimyung University College of Medicine, Daegu, Republic of Korea

*Corresponding author:
Ji Hoe Heo, MD, PhD
Department of Neurology, Yonsei University College of Medicine
50-1, Yonsei-ro, Seodaemoon-gu, Seoul, 03722, Republic of Korea
Tel.: +82-2-2228-1605, Fax: +82-2-393-0705
Email: jhheo@yuhs.ac

Supplementary Methods

Imaging protocol

Patients who were eligible for intravenous tissue-plasminogen activator (IV tPA) were initially evaluated with non-contrast computed tomography (NCCT) and then with follow-up NCCT with CT angiography (CTA) at the end of IV tPA infusion[1]. Patients who were eligible only for intra-arterial reperfusion therapy (IART) initially underwent NCCT and CTA. Thus, all patients underwent NCCT and CTA before IART.

NCCT was performed by a multidetector row CT scanner (LightSpeed Plus; GE Healthcare, Milwaukee, WI). NCCT included thin-section (1-mm or 1.25-mm thickness) as well as conventional 5-mm images, according to a predetermined protocol. Conventional axial 5-mm NCCT was performed first, with the following parameters: 120 kVp; 250 mA; rotation time, 0.8 s; and FOV, 25 cm. Overall, 28–30 images were obtained per examination, according to the size of the patient's head. Helical 1.25-mm or 1-mm NCCT was also obtained after 5-mm CT, with the following parameters: 120 kVp; 250 mA; rotation time, 0.8 s; pitch, 0.75; FOV, 25 cm; matrix, 512 × 512; and pixel size, 0.49 × 0.49 mm. The scan was obtained parallel to the plane of the inferior orbital rim to the basin and ended 7.5 cm above it in order to reduce the radiation dose. All images obtained from 1.25-mm or 1-mm NCCT were reconstructed at 0.6-mm thickness.

Intra-arterial reperfusion therapy

IART was considered in patients who did not respond to IV tPA (Actilyse; Boehringer-Ingelheim, Ingelheim, Germany) or in those ineligible for IV tPA but presented within 8 hours of symptom onset. In case of posterior circulation stroke, IART was allowed up to 12 hours from symptom onset. Stent retriever was the initial endovascular treatment modality of choice since September 2010 except for patients with occlusion of the distal intracranial artery or those with unsuitable angioarchitecture for the approach with stent retriever.

IART procedures were performed by two interventional neuroradiologists with 10 or more years of experience. We used an 8- to 9-F guiding catheter for anterior circulation and 6-F for posterior circulation. Balloon guiding catheter was routinely used for anterior circulation since its

introduction from May 2013. Solitaire (ev3/Covidien, Irvine, CA, USA) was exclusively used for stent retriever thrombectomy. The number of stent retriever passes was not specified in the protocol. If recanalization was not achieved by stent retriever alone, other rescue treatments were performed, which included IA urokinase infusion, aspiration using a Penumbra device, mechanical clot disruption, and stenting. IA abciximab or tirofiban was used in case of reocclusion.

Standardization of Hounsfield Unit (HU)

To determine the thrombus density, the HU of the culprit thrombus was standardized to the HU value of contralateral normal M1 segment of the middle cerebral artery: corrected HU of the thrombus ($HU_{corrected}$ = mean $HU_{contralateral} \times [HU_{ipsilateral} / HU_{contralateral}]$). For occlusions other than middle cerebral artery (MCA), the HU of the normal left M1 segment was used for standardization, because the HUs of the normal basilar artery and MCA are well correlated[2].

References

1. Kim, Y. D. et al. Time-dependent thrombus resolution after tissue-type plasminogen activator in patients with stroke and mice. *Stroke* 46, 1877–1882 (2015).
2. Nam, H. S. et al. Prediction of thrombus resolution after intravenous thrombolysis assessed by CT-based thrombus imaging. *Thromb Haemost* 107, 786–794 (2012).

Supplemtary Table

Supplementary Table S1. Univariable multinomial logistic regression analysis for the number of stent retriever passes.

	First-pass recanalization		2- or 3-Pass recanalization		≥4-Pass recanalization	
	OR (95% CI)	P	OR (95% CI)	P	OR (95% CI)	P
Age, per 1 year	0.98 (0.94–1.02)	0.292	1.00 (0.95–1.04)	0.892	0.97 (0.92–1.02)	0.210
Sex, men	1.03 (0.43–2.47)	0.945	1.42 (0.55–3.65)	0.472	1.85 (0.62–5.46)	0.268
Hypertension	0.51 (0.18–1.43)	0.200	0.76 (0.25–2.35)	0.632	0.67 (0.19–2.35)	0.532
Diabetes	1.06 (0.42–2.71)	0.899	0.76 (0.27–2.17)	0.613	1.48 (0.48–4.55)	0.492
Smoking	2.33 (0.48–11.4)	0.296	1.39 (0.24–8.10)	0.718	5.25 (0.98–28.2)	0.053
Hypercholesterolemia	1.48 (0.48–4.50)	0.493	0.78 (0.21–2.84)	0.704	0.66 (0.14–3.07)	0.591
Initial NIHSS score	0.91 (0.84–0.99)	0.024	0.96 (0.88–1.04)	0.331	1.01 (0.92–1.11)	0.857
Atrial fibrillation	0.80 (0.32–1.98)	0.628	1.09 (0.40–2.95)	0.865	0.79 (0.26–2.40)	0.676
Occlusion sites						
Internal carotid artery	Reference		Reference		Reference	
Middle cerebral artery	1.02 (0.32–3.24)	0.979	2.37 (0.80–6.98)	0.117	2.37 (0.87–6.42)	0.110
Posterior circulation	0.66 (0.12–3.50)	0.625	1.10 (0.24–4.96)	0.901	1.61 (0.43–5.99)	0.475
Use of intravenous tPA	1.65 (0.66–4.15)	0.286	1.76 (0.65–4.74)	0.264	0.86 (0.27–2.80)	0.808
Use of BGC	3.17 (1.26-7.99)	0.014	3.09 (1.14-8.33)	0.026	1.48 (0.48-4.55)	0.492
Size of stent retriever						
4/15	0.41 (0.02-6.78)	0.533	0.68 (0.04-11.3)	0.785	2.70 (0.23-31.9)	0.430
4/20	Reference					
6/30	0.41 (0.02-6.79)	0.533	1.35 (0.12-15.7)	0.810	4.05 (0.39-41.9)	0.240
Time from onset to puncture, per 10 minutes	0.98 (0.96–1.01)	0.116	0.99 (0.97–1.02)	0.666	0.99 (0.96–1.02)	0.427
Thrombus density, per 1 corrected HU	0.99 (0.95–1.04)	0.701	0.96 (0.90–1.01)	0.110	0.99 (0.93–1.04)	0.647
Thrombus volume, per 10 mm^3	0.93 (0.89–0.97)	0.002	0.98 (0.94–1.02)	0.237	0.99 (0.95–1.04)	0.709

Odds ratio was calculated over no recanalization group. OR, odds ratio; CI, confidence interval; NIHSS, National Institutes of Health Stroke Scale; tPA, tissue plasminogen activator; BGC,

balloon guiding catheter; HU, Hounsfield unit.
